# Supplementary figures and images for: Vertical transmission of maternal DNA through extracellular vesicles associates with altered embryo bioenergetics during the periconception period
Source: eLife. 2023 Dec 27;12:RP88008. doi: 10.7554/eLife.88008 (PMC10752591; doi:10.7554/eLife.88008)

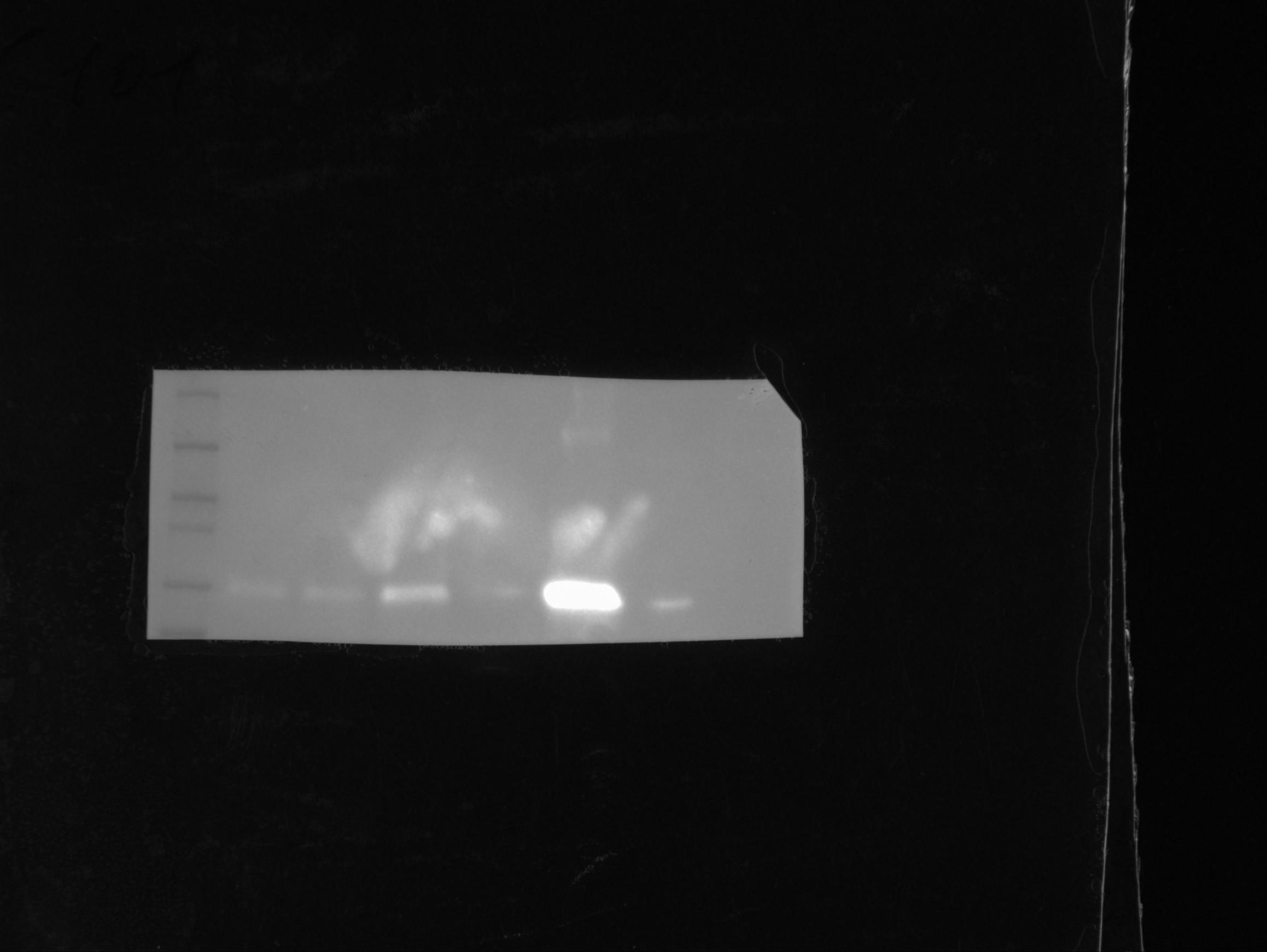

Supplement: Figure 1—source data 1. [file elife-88008-fig1-data1.zip › TSG101.jpg]

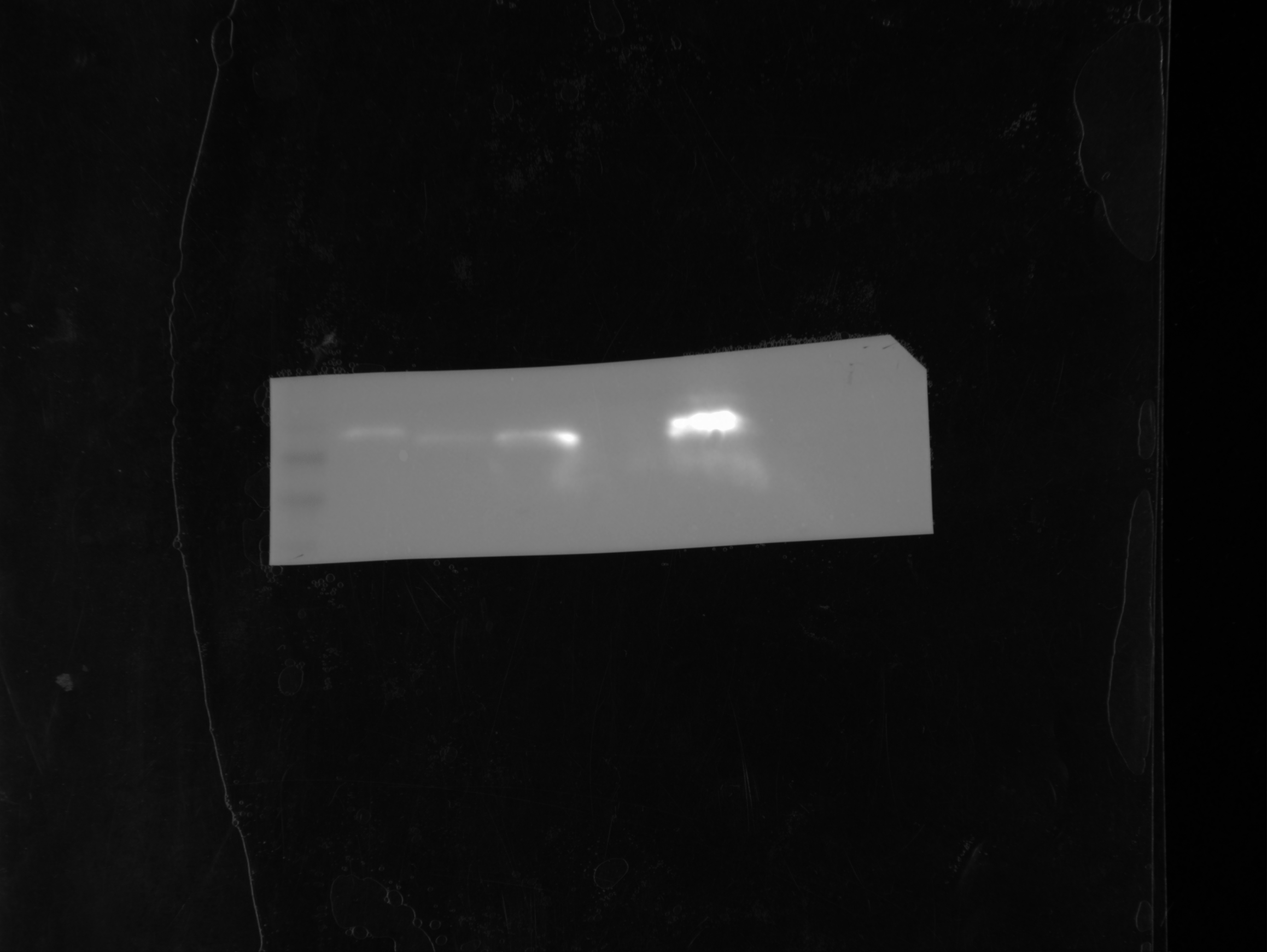

Supplement: Figure 1—source data 1. [file elife-88008-fig1-data1.zip › CD9.tif]

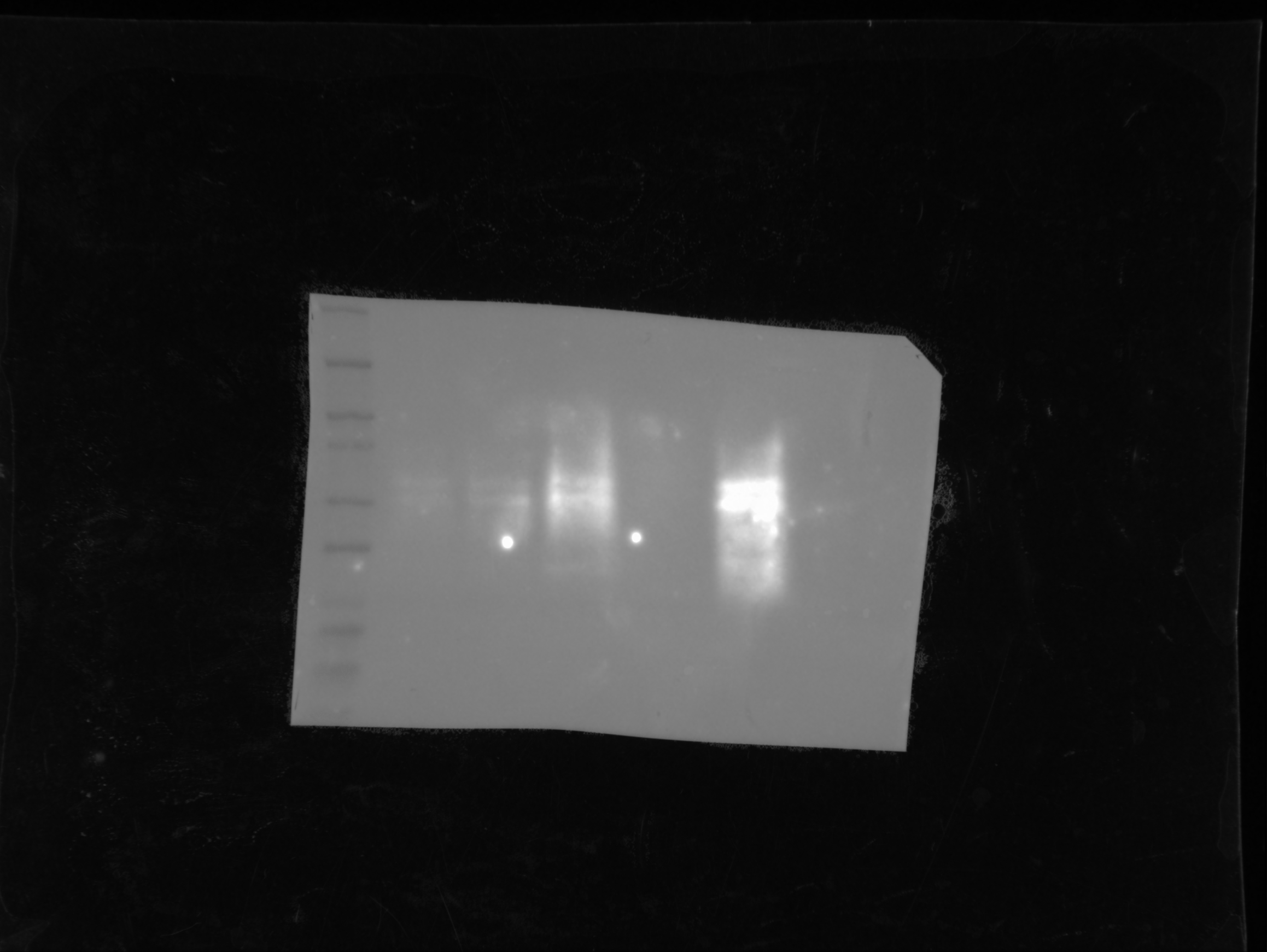

Supplement: Figure 1—source data 1. [file elife-88008-fig1-data1.zip › CD63.tif]
